# Describing the intestinal microbiota of Holstein *Fasciola-*positive and *-*negative cattle from a hyperendemic area of fascioliasis in central Colombia

**DOI:** 10.1371/journal.pntd.0009658

**Published:** 2021-08-09

**Authors:** Angie L. Ramírez, Giovanny Herrera, Marina Muñoz, Laura Vega, Lissa Cruz-Saavedra, Diego García-Corredor, Martin Pulido-Medellín, Diana M. Bulla-Castañeda, Julio Cesar Giraldo, María Consuelo Bernal, José Fernández-Manrique, Luis R. Vásquez-Arteaga, Juan David Ramírez

**Affiliations:** 1 Centro de Investigaciones en Microbiología y Biotecnología–UR (CIMBIUR), Facultad de Ciencias Naturales, Universidad del Rosario, Bogotá, Colombia; 2 Grupo de Investigación en Medicina Veterinaria y Zootecnia (GIDIMEVETZ), Facultad de Ciencias Agropecuarias, Universidad Pedagógica y Tecnológica de Colombia (UPTC), Tunja, Colombia; 3 Grupo de Investigación en Parasitología y Microbiología Tropical, Programa de Biología, Universidad INCCA de Colombia, Bogotá, Colombia; 4 Facultad de Medicina y Ciencias de la Salud, Universidad Militar Nueva Granada, Bogotá, Colombia; 5 Escuela de Ciencias de la Salud (Ecisalud), Universidad Nacional Abierta y a Distancia, Bogotá, Colombia; 6 Laboratorio de Parasitología, Escuela de Ciencias Animales, Facultad de Ciencias Agropecuarias y Recursos Naturales, Universidad de los Llanos, Villavicencio, Colombia; 7 Centro de Estudios en Microbiología y Parasitología, Facultad de Ciencias de la Salud, Universidad del Cauca, Popayán, Colombia; National University of Ireland Galway, IRELAND

## Abstract

The ability to identify compositional changes in the intestinal microbiota of parasitized hosts is important for understanding the physiological processes that may affect animal productivity. Within the field of host–parasite interactions, many studies have suggested that helminths can influence the microbial composition of their hosts via their immunomodulatory effects. Bovine fascioliasis is a helminthiasis widely studied by immunologists, but with little information available regarding gut microbial communities. Thus, we aimed to describe the composition of the intestinal microbiota of Holstein *Fasciola*-positive and -negative cattle using parasitological methods and ELISA (enzyme-linked immunosorbent assay). Bovine fecal samples (n = 65) were obtained from livestock slaughter plants in the Cundi-Boyacense Colombian highlands (a hyperendemic region for bovine fascioliasis) and studied by amplicon-based next-generation 16S-rRNA and 18S-rRNA gene sequencing. From these samples, 35 were *Fasciola hepatica*-negative and, 30 were *F*. *hepatica*-positive in our detection analysis. Our results showed a reduction in the relative abundance of Bacteroidetes and Ascomycota in the *Fasciola*-positive samples, along with decreased relative abundances of the commensal taxa previously associated with fermentation and digestion processes. However, metabolomic approaches and functional analyzes of the intestinal microbiota are necessary to support these hypothesis. These findings are a small first step in the development of research aimed at understanding how microbial populations in bovines are modulated in liver helminth infections.

## Author summary

*Fasciola hepatica*, a liver parasite, infects a wide variety of hosts, mostly ruminants. Ruminant infections with this parasite cause economic losses worldwide, mainly in livestock. Given its importance, much research has been carried out on this parasite, a lot of which has focused on its ability to alter the immune responses of its host. However, little is known about the numerous other interactions it has with the host and how they might affect the host’s intestinal microbiota. Here, we observed a reduction in the abundance of microorganisms associated with the fermentation and digestion of vegetable fiber, two important processes in animal health and productivity. Therefore, it is possible that *F*. *hepatica*, in addition to the previously documented liver alterations, also generates changes in the intestinal microbiota that may impact its bovine host. These findings are a small first step in the research of microbial populations in cattle infected with liver helminths which open the door to further research aimed at identifying alternative treatments for parasites such as *F*. *hepatica*.

## Introduction

Intestinal microbiota in animal species of veterinary interest have attracted research attention because of their involvement in physiological processes such as nutrition, health, and animal productivity [1]. In cattle, the microorganisms present in the gastrointestinal tract are responsible for degrading cellulose and producing volatile fatty acids (VFAs) such as acetate, butyrate, and propionate, which contribute to more than 70% of an animal’s energy [2,3]. Therefore, the conservation of commensal microbial populations is vitally important for maintaining steady homeostasis in an individual [2]. However, the compositional structure of the microbiome can be altered by intrinsic and extrinsic factors, among which diet, host genetics, age, health status, geographic location, and previous infections are primary factors [4]. Recent research suggests that helminths can also alter the composition of the host’s intestinal microbiota via their immunomodulatory effects [5–7]. For this reason, attention in the scientific community has been directed to understanding the factors that can alter microbial populations and, in particular, the mechanisms by which helminths interact with the intestinal microbiota [5].

Most studies conducted in this area, however, have focused on human hosts and murine models, thereby neglecting numerous species of veterinary interest in which helminthiasis is commonly reported. For example, despite the economic importance of ruminants, to the best of our knowledge there are only six studies that have evaluated the impact of gastrointestinal helminths on the composition of a host’s microbiome. The first research paper in this field was published by Li *et al*. in 2011, in which the abomasal microbiota of bovines was evaluated after partial immunization with *Ostertagia ostertagi* [8]. Despite the host-detrimental biology of this parasite, the authors reported that infection with *O*. *ostertagi* did not induce a significant change in the composition of the microbial community in the immunized animals. Conversely, the work carried out in sheep co-infected by the nematodes *Haemonchus contortus* and *Teladorsagia circumcincta* reported an alteration in the abundance of several ovine intestinal bacterial communities that were accompanied by a decrease in alpha diversity after 21 days of infection [6]. Later on, an investigation carried out in 2016 by Li *et al*., found alterations in the abomasal microbiome of *H*. *contortus*-infected goats [9]. These alterations were observed by changes in the relative abundance of approximately 19% of the 432 operational taxonomic units (OTUs) at the species level in the abomasal microbiome. These results were later corroborated by the study carried out by El-Ashram and collaborators, which sought to describe the impact of chronic and acute *H*. *contortus* infections in the abomasal and ruminal communities of sheep [10]. This study, like the one reported by Li *et al.* in 2016, found an increase in the bacterial load of the abomasum and significant changes in the abundance and composition of the abomasal and ruminal microbiota. Later, a study carried out by Mamun *et al* in 2020 provided evidence of differences in the relative abundance of Firmicutes and Bacteroidetes as a consequence of a high load of *H*. *contortus* in sheep, compared to a stable state of certain microbial groups in a low parasite load [11]. Finally, the latest research in this field was conducted by Cortés *et al*. (2020) where the fecal microbiota profiles of sheep that were vaccinated and later infected with *Teladorsagia circumcincta* were compared [12]. The findings from this study revealed associations between the expansion of populations of pro-inflammatory bacteria and the presence of the parasite, as well as longitudinal changes in the abundance of certain taxa such as Bacteroidales RF16, the Porphyromonadaceae family, and the *Porphyromonas* genus. All of these studies, however, have focused on the role of intestinal helminths while neglecting the impact of extra intestinal helminths which, as part of the same system, interact with their host [5,13].

Bovine fascioliasis, a highly prevalent veterinary disease, causes great economic losses worldwide [14]. The organisms responsible for this disease are helminths of the *Fasciola* genus, which live and reproduce sexually in the bile ducts of mammals, generally bovines [15,16]. This parasite inflicts mechanical damage in the liver parenchyma, which lead to weight loss, anemia, reduced fertility, milk production losses (up to 15%), and poor work capacity in infected cattle [11,17,18]. Once the adult stage has developed, *Fasciola* eggs are released into the intestine from where they are excreted in the feces of the definitive host. Thus, the ability to detect *Fasciola* eggs is crucial to the treatment of susceptible animals [19].

In Colombia, bovine fascioliasis causes annual losses exceeding US $ 12,483 in the regions of Antioquia, Boyacá, Cundinamarca, Nariño, and Santander [20,21,22]. Some studies suggest that Boyacá and Cundinamarca are endemic for this disease because they are places that favor the life cycle of the parasite [23]. Giraldo-Forero *et al*. (2016) reported a bovine fascioliasis seroprevalence of 39.4% in Cundinamarca, while Pereira *et al*. (2020) pointed to an infection frequency of 23% in the Boyacá region; hence, bovine fascioliasis imposes a high economic burden of veterinary disease on these areas [21,24]. Despite it is considered a parasite of great interest, research on *Fasciola* has primarily focused on its immunomodulatory effects, leaving many other aspects of host–helminth biology neglected, particularly the gut microbiota [5,25]. Therefore, the present study aimed to describe the composition of the intestinal microbiota of Holstein *Fasciola*-positive cattle (*Bos taurus*) from the Cundi-Boyacense Colombian highlands through an amplicon sequencing approach on 16S-rRNA and 18S-rRNA genes.

## Methods

### Ethics statement

This study was approved by the ethics board from the Pedagogical and Technological University of Colombia (UPTC is its Spanish acronym) under Act number 06 from 2019. This project is considered of minimum risk under the Colombian resolution of the ministry of health 8430 from 1993. Fecal samples were collected from Holstein cattle from livestock slaughter plants ensuring the animal welfare.

### Sample collection and *Fasciola* identification

Altogether, 65 fecal samples from Holstein cattle were collected from livestock slaughter plants located in the Colombian Cundi-Boyacense highlands (a region hyperendemic for bovine fascioliasis). Initially, the presence of adults in bile ducts or *F*. *hepatica* eggs in the bile contents was checked *in situ*. Fecal and blood serum samples were taken and transported to the laboratory of the Research Group in Veterinary Medicine and Zootechnics (GIDIMEVETZ is its Spanish acronym) of the Pedagogical and Technological University of Colombia (UPTC) for the respective parasitological analyzes. These analyzes involved *Fasciola* eggs identification using the formol-ether concentration technique on fecal matter, as well as the identification of other helminths and/or protozoa that may be found in coinfections with *Fasciola* (S1 Table) [26]. Additionally, to confirm exposure to the parasite in the samples to be analyzed, we performed an in-house enzyme-linked immunosorbent assay with self-antigen from the blood serum samples [27]. Based on these experiments, the presence of the *F*. *hepatica* parasite was detected by at least one of the tests (serological or parasitological analysis). Thus, 30 positive and 35 negative samples were used to analyze the *Fasciola* status of the cattle (S1 Table).

### DNA extraction and library preparation

Genomic DNA was extracted from the 65 fecal samples using the Stool DNA Isolation Kit (Norgen, Biotek Corporation, Sacramento, CA, USA) following the manufacturer’s instructions. The concentration of the extracted DNA was evaluated using the NanoDrop ND-1000 spectrophotometer, and the 260/280 absorbance ratio was calculated to determine the quality. DNA integrity was evaluated using 1% agarose gel electrophoresis. The samples that met these quality parameters were sequenced by Novogene Corporation (Sacramento, CA, USA), using the Illumina Novaseq 6000 system. Mate-paired libraries were constructed using paired-end sequencing to generate amplicons of the V4 hypervariable region of the 16S-rRNA and 18S-rRNA genes using 515F-806R and 528F-706R primers as previously reported (150 bp and 350 bp, respectively)[28,29]. As part of the technical service, Novogene verified the quality scores for DNA purity using Nanodrop (OD260/OD280 ratio), DNA degradation tests or potential contamination using agarose gel electrophoresis, and the DNA concentration was quantified using Qubit 2.0. To further ensure the quality of each analysis, primers and adapters, reads with lengths <60-bp (after primer and adapter trimming), reads containing N >10% (N represents an undetermined base), and reads with Q scores < = 5 (over 50% of the total bases), were all removed.

### Taxonomical assignment and bioinformatic analyzes

We next performed quality control on the sequencing reads obtained by FASTQC and MULTIQC, taking into account a quality score greater than 30 [30,31]. The paired-end demultiplexed Illumina sequencing reads were imported into DADA2 where a table of amplicon variants (ASVs) was generated, a term analogous to OTUs, where each sequence variant is considered unique even if it differs from another by a single nucleotide [32,33]. The taxonomic assignments were then made from the ASVs we identified, making use of the SILVA v136 and Protist Ribosomal Reference database (PR2) v4.12.0 to analyze the 16S and 18S sequences, respectively [34,35]. The databases were built in-house from the representative sequences of each molecular marker registered in GenBank and were used to make taxonomic assignment of sequences not identified by SILVA and PR2. These sequences and databases were imported into BLAST, with the minimum parameters of 90% identity and e-values of 10, from where we made the taxonomic assignments. Once the taxonomic assignments had been made, the sequences were imported into the DADA2 pipeline in R software (Phyloseq and Vegan packages) where relative abundances, alpha diversity, and beta diversity analyses were made [36]. Rarefaction curves were also generated to corroborate that the sequencing depths were sufficient to cover the microbial diversity in each sample (S1 and S2 Figs).

In addition to the parasitological analysis, to evaluate possible coinfections, we filtered the reads corresponding to nematodes and platyhelminthes in the database of taxonomic assignments generated by PR2. Thus, samples in which the presence of parasite DNA was identified with a relative abundance of at least 10% (with respect to the total number of reads) were considered positive for co-infections. Finally, we generated a correlation analysis between ASVs corresponding to bacterial genera and eukaryotic ASVs to determine correlations between these and potential changes in *F*. *hepatica* states (positive or negative).

### Statistical analyses

An unsupervised principal coordinate analysis (PCoA) based on the Bray-Curtis dissimilarity index was used to group the samples by their infection status. Additionally, the association between the microbiota composition and the *Fasciola* status was investigated using a multivariate analysis of covariance (MANCOVA) and the distance matrices (Adonis) in R software [11]. The alpha diversity analyzes were evaluated using Shannon index, the derivation of the Simpson (1-D) diversity index (which will be referred to from now on as Simpson’s index to facilitate its interpretation) and the richness observed from the ASVs obtained per sample. To compare the relative taxa abundance belonging to the intestinal microbiota of bovines in the *Fasciola* states, the Mann-Whitney U test was used [37]. For the correlation analysis, a Benjamin-Hochberg correction was used to reduce type I errors and false positive rates using corrplot and psych packages [38,39]. To test the differences in bacterial taxa between *Fasciola*-positive and -negative cattle, Analysis of Composition of microbiomes (ANCOM) was carried out using R software [40,41]. Considering that ANCOM makes comparisons of the microbial composition between populations on the basis of taxonomic operations units (OTUs), term in which genomic sequences are clustered by similarity, we generated a table analogous to the table of abundances of OTUs by samples grouping the sequences taxonomically assigned to the same genus [40] (S3 and S4 Tables). However, since in these abundance tables, the excess of zeros presents a challenge when comparing the groups, as part of the preprocessing before performing ANCOM, we added a pseudo-count to the table of abundances of OTU per sample as part of a common strategy to handle these zeros as previously reported [40,42]. Likewise, to avoid spurious results, we corroborated changes in the microbial composition between groups using ANOVA-like differential expression (ALDEx) in R (version 4.1.0) as reported elsewhere [43,44]. The significance level used for all the statistical tests was 0.05. Finally, we performed a linear discriminant analysis (LDA) effect size (LEfSe) using the Galaxy/HutLab online tool (https://huttenhower.sph.harvard.edu/galaxy/) to determine changes in microbial biomarkers, which are defined as indicators of biological states, from changes in their relative abundances between *F*. *hepatica* states [45]. The parameters used were an alpha value from the Kruskal-Wallis factorial test between classes of 0.05, and a logarithmic LDA score threshold for discriminatory characteristics of 2.0.

## Results

### Processing the Illumina reads

The statistics generated by multiqc showed that all the reads had a good quality average (Phred score> 27) for both 16S and 18S. Going deeper, we obtained an average of 10,068 reads per sample, with a minimum of 5,126 and a median of 10,016 reads per sample (SD = 813) for the case of 16S. Regarding 18S, we obtained an average of 4,117 reads per sample, with a median of 4,098 and a minimum of 4,098 reads (SD = 1102) per sample.

Altogether, 49,570 ASVs were identified using state-of-the-art sequencing on the 16S-rRNA gene amplicons from the DNA extracted from the fecal samples of the bovines studied herein. These ASVs were assigned to 382 bacterial genera and four Archaean genera. In contrast, 4,802 ASVs were identified in the 18S-rRNA gene amplicons from the same samples. These ASVs were filtered to exclude any taxa that lacked biological sense (microorganisms that are not part of the composition of the bovine intestinal microbiota and correspond to non-specific taxonomic assignments from the reference database, such as environmental microorganisms) in this study (S2 Table). Thus, the 3,555 ASVs that we included made biological sense for the R software analysis. Rarefaction curves were then generated for each molecular marker (16S and 18S), and this showed that the sequencing depth was enough to reveal the diversity of prokaryotic, archaea, and eukaryotic communities (S1 and S2 Figs).

To avoid confusion, from now on, the samples will be named in the non-consecutive order described in S1 Table.

### Relative abundances of prokaryotes by *Fasciola* status

**Fig 1:**
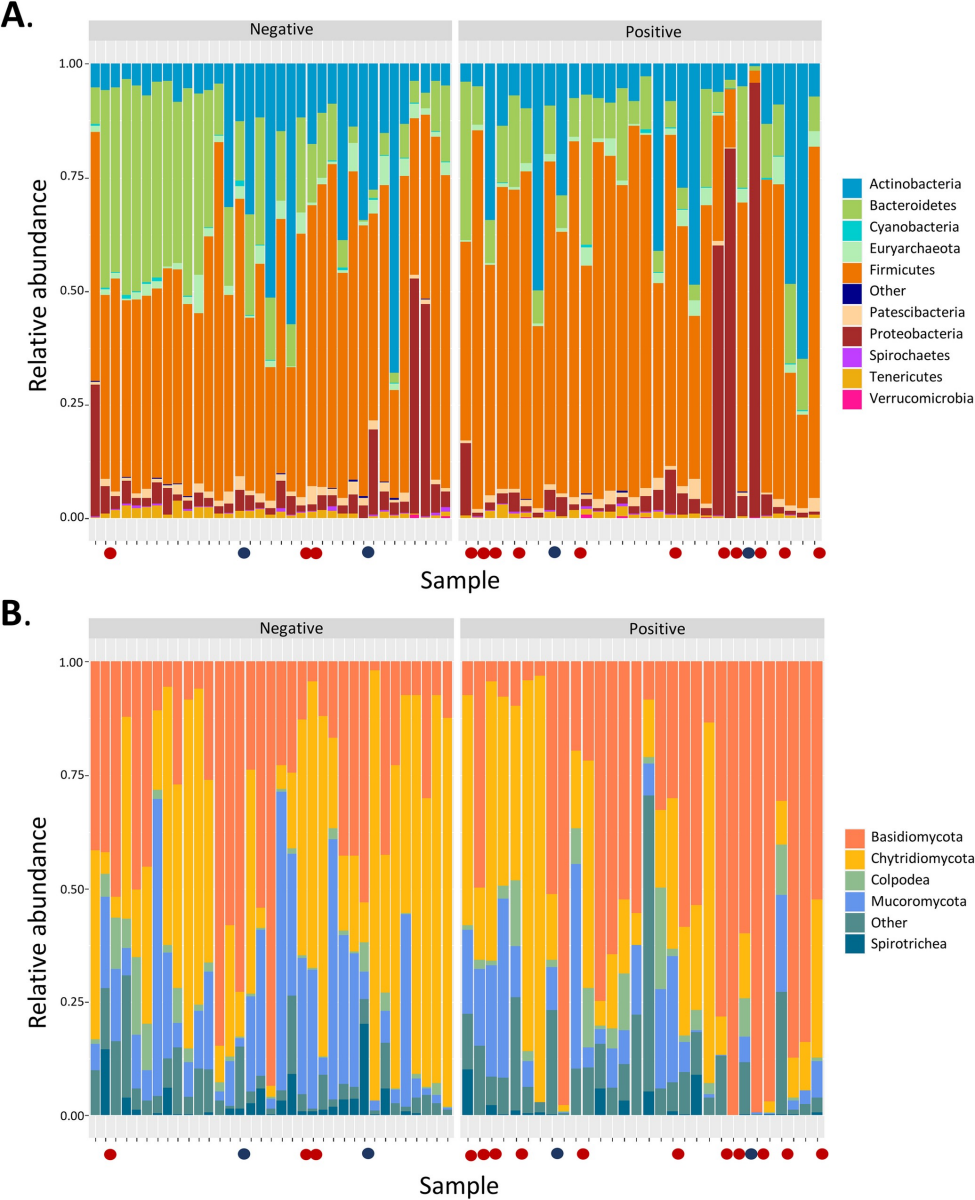
Relative abundances of the most representative prokaryotic (A) and eukaryotic (B) phyla from the intestinal microbiota of *Fasciola*-negative and *Fasciola*-positive cattle. The circles at the bottom of each sample indicate the coinfections detected by parasitological analysis (red) and next-generation read identification in the samples (blue). For the 16S gene, the category “others” contains the following phyla: Acidobacteriota, Campylobacteriota, Chloroflexi, Cyanobacteria, Deinococcota, Dependentiae, Desulfobacteroidota, Elusimicrobiota, Fibrocateriota, Fusobacteriota, Gemmatimonadota, Halacoccotamo, Nitrospirota, and Symrostanergotta. For the 18S gene, the category “others” contains the following classes: Apusomonadidae, Bicoecaea, Bastocladiomycota, Breviatea, Chlorophyta, Choanoflagellatea, Coccidiomorphea, Colpodea, Colpodellidea, Colponemea, Cryptomycota, Dictyochophyceae, Eugleomyyphenozoin, Filosebytestriida, Gregaromyrinhophethyyphethyobia, Filosethulosa, Gregarophyterolho-phecyphozoin, Filosethrocetam Lithostomatea, Lobosa, Mucoromycota, Mycetozoa, Nassophorea, Nemátoda, Oligohymenophorea, Oomycota, Opalinata, Palmophyllophyceae, Platyhelminthes, Spirotrichea, Syndiniales, Tubulinea, Variosea, and Zoopagomycota.

**Fig 2:**
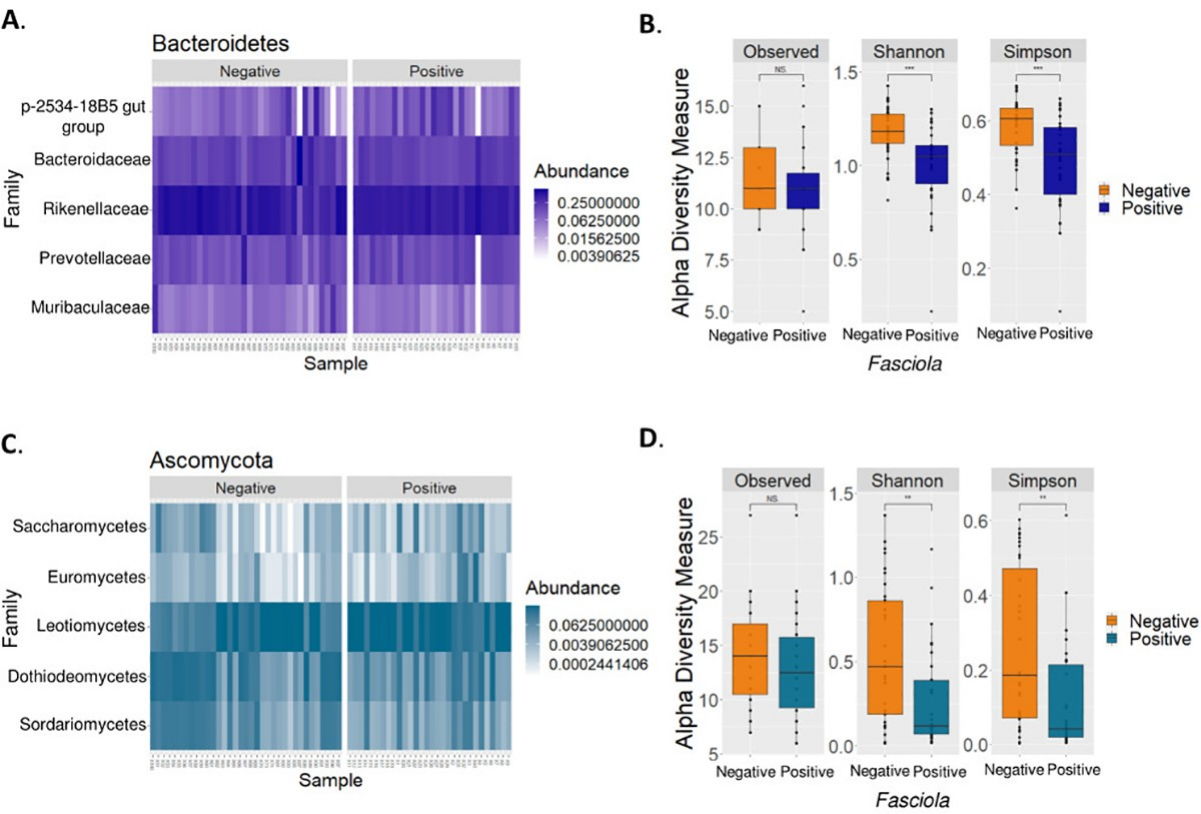
(A) Heatmap of the relative abundances at family level for the Bacteroidetes phylum and (C) for the Ascomycota phylum among the *Fasciola*-positive bovine group and the *Fasciola*-negative group. (B) Boxplot of the diversity indices (Shannon, Simpson (1-D), and Observed) for prokaryotes and (D) for eukaryotes that distinguish between the *Fasciola* states.

Firmicutes (52.1%), Bacteroidetes (21%), and Actinobacteria (12.9%) phyla were the most abundant in all samples (Fig 1A), followed by Proteobacteria (6.1%), Euryarchaeota (2.68%), Patescibacteria (1.8%) and Cyanobacteria (0.5%). Of these phyla, only Bacteroidetes showed a statistically significant reduction in the *F*. *hepatica*-positive samples (Mann-Whitney-Wilcoxon test, *p*-value = 0.0266). We were struck by the fact that some, but not all, *F*. *hepatica*-negative samples showed increased Bacteroidetes levels within the same *Fasciola* infection state (Fig 1A). At the lower taxonomic level, the generated heatmap showed that the *Bacteroidaceae*, *Rikenellaceae*, *Muribaculaceae*, and *Prevotellaceae* families (Fig 2A) were the most representative of the Bacteroidetes phylum, with a reduction in *Bacteroidaceae* (Mann-Whitney-Wilcoxon test, *p-*value = 0.04716) and *Rikenellaceae* (Mann-Whitney-Wilcoxon test, p-value = 0.03639) in the *F*. *hepatica*-positive cattle. Therefore, we evaluated the changes occurring at the relative abundance level for the Firmicutes phylum. This involved comparing the ASVs corresponding to the most abundant families among the *Fasciola* states (S4 Fig). We found that *Lachnospiraceae*, *Peptostreptococcaceae* and *Carnobacteriaceae* were the families with the highest relative abundances for both groups (S3 Fig). However, statistically significant differences in the *Fasciola*-positive bovine group were only seen with the latter two families, with a reduction in *Carnobacteriaceae* (Mann-Whitney-Wilcoxon test, p-value = 0.04138), and an increase in *Peptostreptococcaceae* (Mann-Whitney test, Wilcoxon, *p-*value = 0.009438).

We next compared the samples by *Fasciola* status in terms of their diversity levels based on the observed richness and the Shannon and modified Simpson (1-D) indices (Fig 2B). The analysis identified statistically significant reductions in richness (Shannon index p-value = 0.00014) and dominance (Simpson index p-value = 0.00085) for the prokaryotic microorganisms belonging to the *Fasciola*-positive bovine group. This pattern is possibly associated with the decrease found at the level of microbial members belonging to the Bacteroidetes and Firmicutes phyla.

The analysis of composition of microbiome (ANCOM) in prokaryotic genera identified 385 taxa from which *Solobacterium* (W = 355), *Paeniclostridium* (W = 354), *Parabacteroides* (W = 351) and *Allorhizobium-Neorhizobium-Pararhizobium-Rhizobium* (W = 351) showed significant differences (p < 0.05) in abundance between states. *Paeniclostridium* and the genus *Allorhizobium-Neorhizobium-Pararhizobium-Rhizobium* were relatively abundant in the *Fasciola*-positive group while the abundance of the genus *Solobacterium* and *Parabacteroides* were increased in the *Fasciola*-negative group (S5 Fig).

### Relative abundances of eukaryotes by *Fasciola* status

Our relative abundance analysis showed that the highest proportion of microbes we detected corresponded to the Ascomycota phylum (25.9%) followed by Basidiomycota (10.3%), Chytridiomycota (2–8%), Colpodea (2.1%) and Mucoromycota (1.4%) (S3 Fig). However, because the abundances of the other taxonomic groups were not clear, we generated a relative abundance plot (excluding Ascomycota) (Fig 1B). Nevertheless, no statistically significant differences were observed except for Ascomycota where there was a reduction in the *Fasciola*-positive bovine group (Mann-Whitney-Wilcoxon test, p-value = 0.01007). Hence, we compared the abundances among the *Fasciola* states to identify the most representative families of the main phyla (S4 Fig). We identified statistically significant changes in Ascomycota, the families of which corresponded to *Leotiomycetes*, *Dothideomycetes*, *Sordariomycetes*, *Saccharomycetes* and *Eurotiomycetes* (Fig 2C); all of them showed statistically significant reductions except for *Leotiomycetes*, where we observed an increase in the *Fasciola*-positive state (Fig 2C). In terms of diversity, we observed that the *Fasciola*-positive bovine group was reduced in richness and dominance of eukaryotic microbial members (Fig 2D) when taking into account the reference Shannon diversity index (Mann-Whitney-Wilcoxon test, p- value = 0.001548) and the Simpson diversity index (Mann-Whitney-Wilcoxon test, p-value = 0.002852).

Considering the differences in Eukaryotes that were observed in the relative abundance analysis, we performed the analysis of composition of microbiome (ANCOM) to identify key eukaryotic genera discriminating the infection status. From the 658 taxa at the genus level, *Pseudozyma* (W = 657), *Neocallimastix* (642), *Pilobolus* (W = 624), *Apiospora* (W = 604), *Cyllamyces* (W = 604), *Leptosphaeria* (W = 600), *Buxtonella* (W = 597), and *Cystobasidiomycetes_X* (W = 597) had higher abundance in the *Fasciola*-positive group of cattle. Among which, it was corroborated by ALDEx that *Pseudozyma* presented a higher abundance in the *Fasciola-*positive group of cattle (S7 Fig). Meanwhile, *Rhodosporidium* (W = 641), *Hyphozyma* (W = 638), *Talaromyces* (W = 637), *Thelebolus* (W = 615) and *Filobasidium* (W = 607) were more abundant in the *Fasciola*-negative bovine group compared to the *Fasciola*-positive one (S6 Fig). Among which, we found by ALDEx that *Filobasidium* and *Talaromyces* presented a higher abundance in the *Fasciola-*negative group (S7 Fig).

### Analysis of beta diversity by infection state

**Fig 3:**
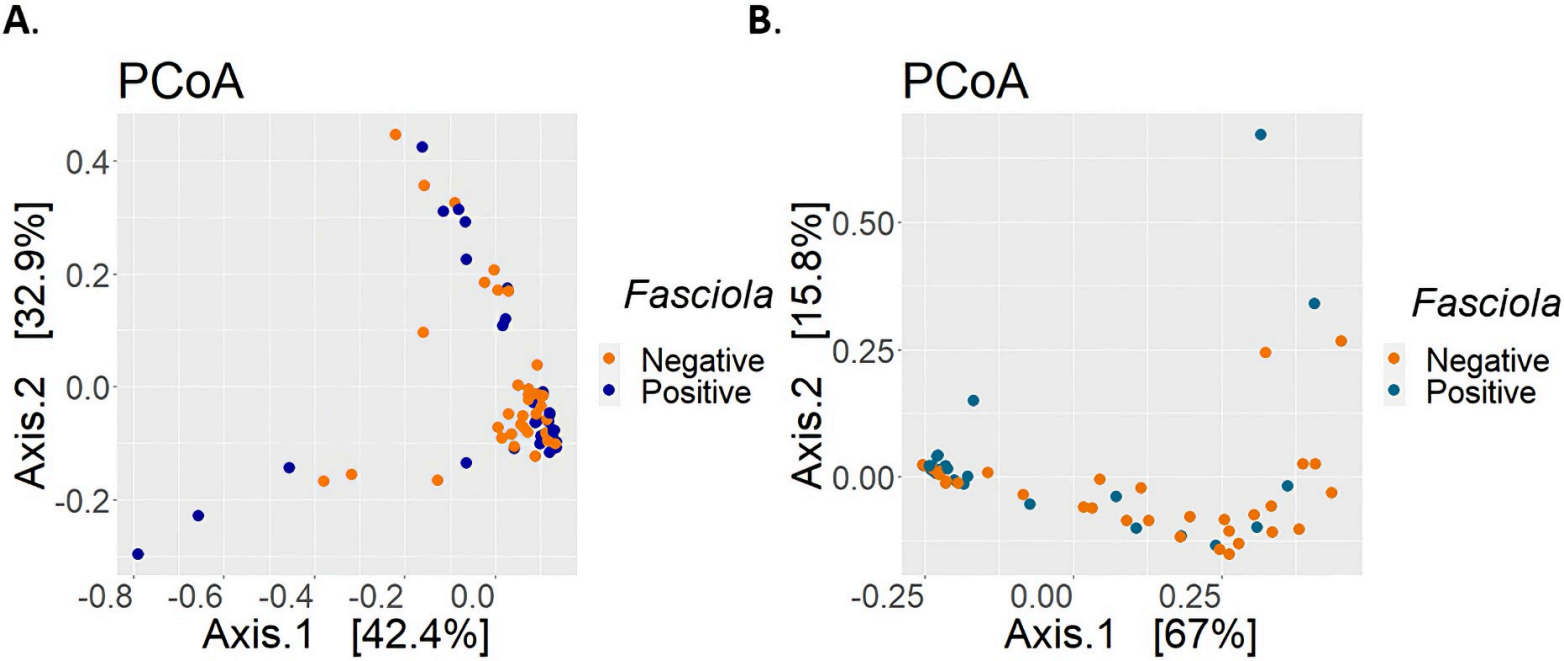
Analysis of the principal coordinates by Bray-Curtis distance of the microbial communities of *Fasciola*-negative and *Fasciola*-positive cattle. (A) PCoA for analysis of prokaryotic microbial communities. (B) PCoA for analysis of eukaryotic microbial communities.

The profiles of the fecal microbiota from the bovine samples were obtained and grouped by *Fasciola* status using a PCoA in which no clear groupings were observed for the microorganisms identified by 16S-rRNA (MANCOVA p-value = 0.796., Fig 3A). However, a statistically significant grouping was obtained for 18S-rRNA (MANCOVA p-value = 0.021., Fig 3B), thereby revealing that the eukaryotic microorganisms were differentiated by *Fasciola* status.

### Biomarkers between *Fasciola* states: LDA effect size (LEfSe)

**Fig 4:**
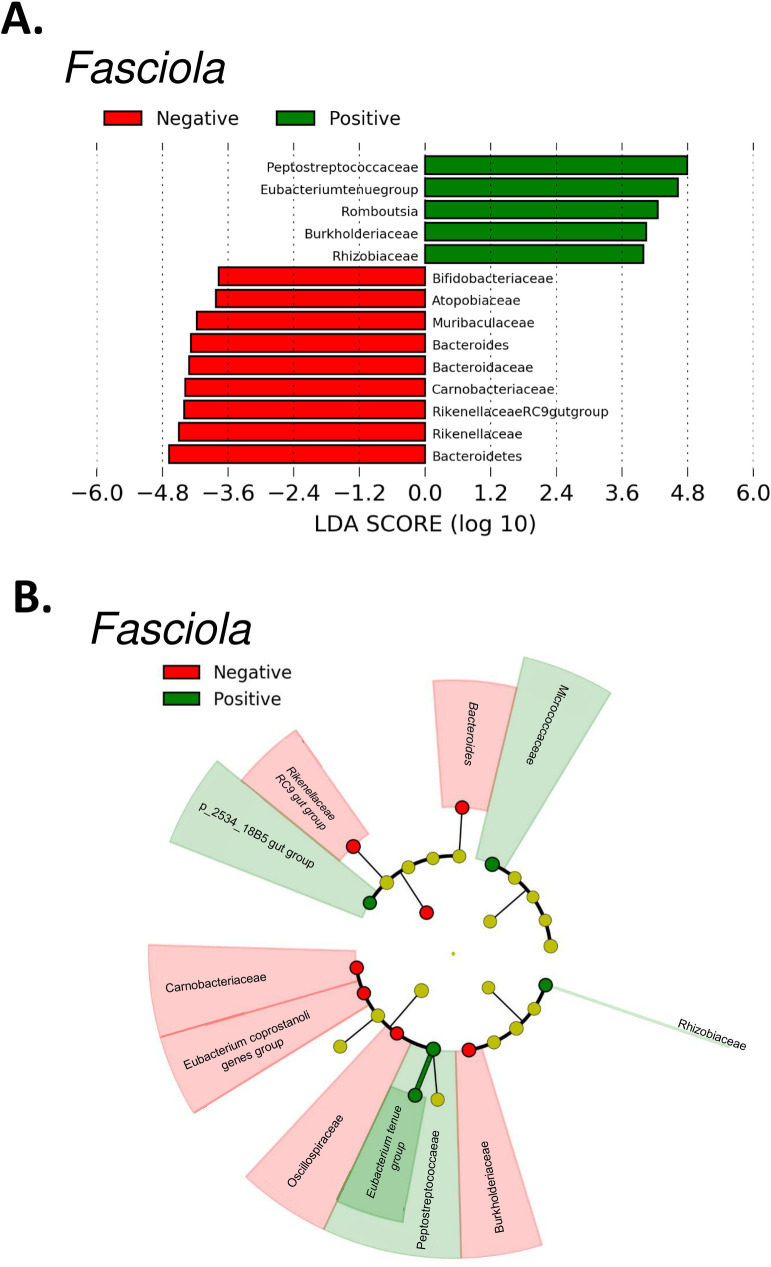
LEfSe analysis for the identification of differentially abundant taxa between *Fasciola* states. (A) Histogram showing the different taxa between the *Fasciola*-positive bovine group and the *Fasciola*-negative bovine group as classified by the effect sizes. (B) Taxonomic representation of statistically and biologically different taxa between the two infection states.

Recent investigations have revealed alterations in the gastrointestinal microbial communities as a consequence of helminth infections in the host [5,46]. We examined these changes at the relative abundance level by *Fasciola* states using LEfSe. Altogether, 14 biomarkers were obtained that showed biologically consistent differences between the infection states (Fig 4A). The taxonomic groups that explained the greatest differences between the samples were the phylum Bacteroidetes for the negatives and the family *Peptostreptococcaceae* for the positives (Fig 4B). In the group of *F*. *hepatica*-positive cattle, *Peptostreptococcaceae* stood out by presenting an LDA score of more than four orders of magnitude, thereby reflecting the increase in its relative abundance. In this same group, the Burkholderiaceae and Rhizobiaceae families and the *Romboutsia* genus and *Eubacterium tenue* group were increased in number compared with those of the negative state (Fig 4A). Regarding the group of *F*. *hepatica*-negative samples, we found nine biomarker taxa corresponding to six families and two genera. At the family level, the differentially abundant bacteria were represented by *Bifidobacteriaceae*, *Atopobiaceae*, *Carnobacteriaceae*, *Rikenellaceae*, *Muribaculaceae*, and *Bacteroidaceae*, whereas a relative abundance reduction for genera was found for the *Rikenellaceae* RC9 gut group and Bacteroides with respect to the group of positive samples.

### Correlation analysis

**Fig 5:**
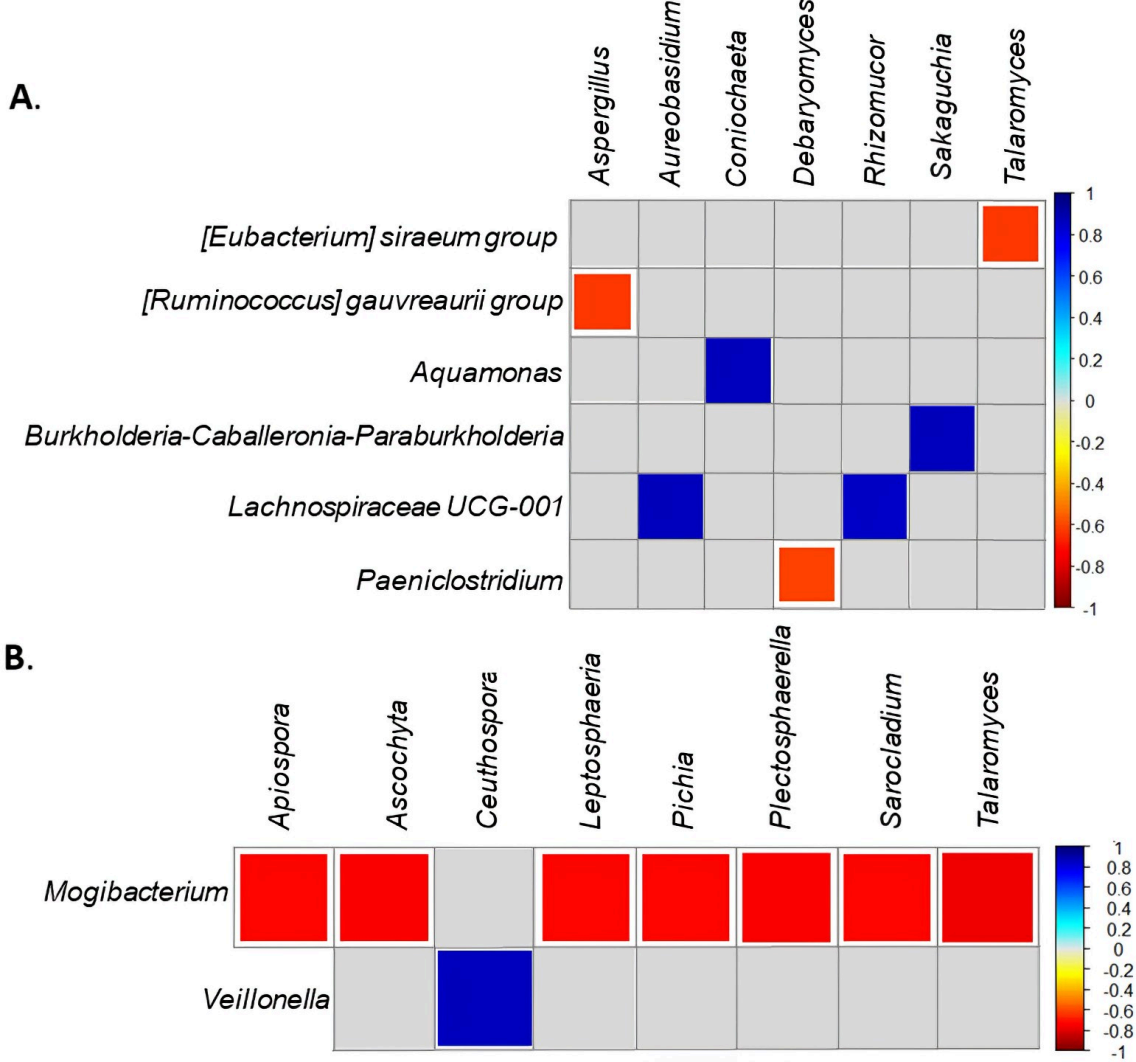
Analysis of the correlations observed between prokaryotic and eukaryotic members of the bovine intestinal microbiota by *Fasciola* state. *Fasciola*-positive bovine group (A), *Fasciola*-negative bovine group (B). Red cells represent a statistically significant positive correlation (*p-*value < 0.05) between genera, while the blue ones represent a statistically significant negative correlation. Cells in which we did not find correlations were filled with grey.

Positive and negative associations were observed in both *F*. *hepatica* negative and positive bovine samples; however, a greater number of associations were obtained when the parasite was present. In the latter, *Aquamonas* and *Coniochaeta*, *Burkholderia-Caballeronia-Paraburkholderia* and *Sakaguchia*, *Lachnospiraceae* UCG-001 and *Aureobasidium*, *Lachnospiraceae* UCG-001 and *Rhizomucor* were positively related. In this same group, negative correlations were found between the *E*. *siraeum* group and *Talaromyces*, the *Ruminococcus gauvreaurii* group and *Aspergillus*, and *Paeniclostridium* and *Debaryomyces* (Fig 5A). Regarding the group *F*. *hepatica*-negative cattle, a negative correlation was observed between *Mogibacterium* and *Apiospora*, *Ascochyta*, *Leptosphaeria*, *Pichia*, *Plectosphaerella*, *Sarocladium* and *Talaromyces*, and a positive correlation was observed between *Veillonella* and *Ceuthospora* (Fig 5B).

## Discussion

To the best of our knowledge, this is the first study to describe the intestinal microbial diversity and abundance in the presence of infection states of *Fasciola*. Herein, the results revealed changes in the relative abundance levels of commensal microbial groups in the *Fasciola*-positive bovine samples (Fig 4A), which is consistent with the findings from investigations carried out in helminth infection scenarios in ruminants [10,47]. However, it is worth clarifying that, although the findings suggest a reduction in the digestion capacity of plant fiber due to alterations in the main commensal genera associated with these functions, this study focused on describing the microbiota in both infection states. Therefore, future metabolomic and mechanistic approaches, and functional analyzes of the intestinal microbiota are necessary to support our hypothesis.

As in other investigations, we found that the most representative taxonomic groups in the bovine intestinal microbiota were Firmicutes, Bacteroidetes and Actinobacteria (Fig 1A) [1,3]. In the Firmicutes phylum, the dominant taxa were *Lachnospiraceae*, *Peptostreptococcaceae* and *Carnobacteriaceae*, which all play important roles in ruminal fermentation, as well as in other digestion-associated processes (S3 Fig) [48,49]. However, our LEfSe analysis pointed towards a reduction in the relative abundance of commensal taxa such as *Carnobacteriaceae* (LDA score> 3.6) in the *Fasciola*-negative cattle group, which might suggest a reduction in ruminal fermentation capacity associated with the function of these microorganisms that should be evaluated in future studies [5,49]. The same analysis revealed *Peptostreptococcaceae* as a potential biomarker for the positive status of *F*. *hepatica*, due to the increased relative abundance of this family in this group (Fig 4A). *Peptostreptococcaceae* is a family of anaerobic bacteria considered commensal because they produce acetate as a fermentation product; hence, their role during infection with this helminth is unclear [50]. Nevertheless, an increased relative abundance of this family has been previously reported during infection with *Hymenolepis diminuta* in mice, in which alterations in the cecal microbiome (mainly at the Firmicutes phylum level) were observed in the presence of the helminth [51]. The mechanism by which the presence of this helminth favors this increased relative abundance is unclear.

Bacteroidetes is a prevalent phylum of bacteria in animals with diets with a high content of plant fiber [52,53]. At the intestinal level, they are involved in the release of short chain fatty acids, which are easily absorbed by the host and are destined for the energy production of the animal. Therefore, changes in the diversity and abundance of members belonging to this phylum can affect intestinal function, impacting bovine health and production [52,54]. Herein, we found an increased relative abundance of Bacteroidetes in some samples within the *Fasciola*-negative group (Fig 1A). Although this pattern was not consistent with any of the variables we evaluated, the ANCOM analysis showed an increase in the abundance of *Parabacteroides* (S5 Fig), a genus belonging to the Bacteroidetes phylum in the *Fasciola*-negative group (S5 Table). Since this was only observed for one genus, further studies are needed to clarify its causality. At the compositional level of the bovine intestinal microbiota, we also found a reduction in the diversity, dominance, as well as relative abundance of commensal families belonging to this phylum (Fig 2A) in the *Fasciola*-positive group. Among them, *Rikenellaceae* and *Bacteroidaceae* stand out for presenting statistically significant reductions in the group of positive cattle for *F*. *hepatica* infection state that was corroborated by the LEfSe analysis, where we found statistical and biological differences in the *Rikenellaceae*, *Muribaculaceae*, and *Bacteroidaceae* families (Fig 4B). This might suggest that when cattle are exposed to this parasite, they might experience a lower capacity to produce VFAs, as well as a lower ability to degrade mucins, both of which are commensal functions associated with *Bacteroidaceae* and *Rikenellaceae*, respectively [55,56]. Nevertheless, this pattern may not necessarily be a consequence of the presence of *Fasciola*; therefore, future approaches are needed to elucidate their role in the production capacity of VFAs and mucins in ruminants naturally infected with *Fasciola* and/or other parasites.

Intestinal fungal populations of ruminants stand out for their high capacity to produce excretory enzymes with cellulolytic, hemicellulolytic, glycolytic and proteolytic activities [8]. Considering their importance, we evaluated the gastrointestinal communities of fungi and their relative abundances between infection states. In general, our division-level analysis showed that the highest fungal proportions detected included Ascomycota, Basidiomycota and Chytridiomycota (Fig 1B). Notably, under the *F*. *hepatica*-positive status, only in Ascomycota was a statistically significant decrease observed. Coincidentally, we obtained significant reductions in microbial diversity, a finding possibly associated with the general decrease in microbial members belonging to this phylum (Fig 2D). Considering that the proportions of eukaryotes are lower than those of prokaryotes in the gut, it is possible that the loss of these microbial groups could facilitate an increased abundance or even the establishment of other microbial communities that differ in structure from the healthy state (Fig 3B). With few studies having considered eukaryotic diversity, this result requires further evaluation. However, Ascomycota is known to be a natural constituent of livestock microbiota, where most of its families secrete cellulose and hemicellulose degrading enzymes [57]. Within this phylum, the five most abundant families presented statistically significant reductions with the exception of Leotiomycetes, where an increase in the infected state was observed (Fig 2C). The above was corroborated by the ANCOM analysis, where *Hyphozyma* (a genus belonging Leotiomycetes), showed a significant increase in abundance of the *Fasciola-*positive group (S6 Fig). The mechanism that explains is unknown for this family; however, there is a family of saprophytic and endophytic fungi of plants and soil suggesting the pivotal need to unveil the specific roles of these taxa in the gut ecosystem [58].

As part of the same physiological system, intestinal microbial communities interact with each other and with their host to maintain a homeostatic state [3]. We evaluated potential correlations between ASVs corresponding to eukaryotes and prokaryotes by *Fasciola* status. Within the group of uninfected cattle (Fig 5B) we observed a negative correlation between *Mogibacterium* and several fungi previously associated with plant fiber digestibility [47]. Interestingly, *Mogibacterium* has been involved in the assimilation of ammonia, whereas another study points to a negative correlation between the abundance of this group and the loss of body mass in humans, then, its role in the intestine is not clear [45,51]. To the best of our knowledge, there is no consensus on the role of *Apiospora*, *Ascochyta*, *Leptosphaeria*, *Pichia*, *Plectosphaerella*, and *Sarocladium* in the gut microbiota of bovines. In addition, most of them are reportedly phytopathogens, then future investigations are needed to confirm their role at the gastrointestinal level and explore if this effect is a consequence of *Fasciola* infection [49,59]. *Veillonella* is a genus associated with lactate fermentation, a precursor of the VFAs, and, as far as we can tell, this is the first study to report on its presence and correlation with a bacterial genus [52]. Nevertheless, based on the type of correlation found herein with a commensal genus, it should be involved in plant material processing.

The correlations found in the group of *Fasciola*-positive cattle (Fig 5A) can mainly explain the presence of commensal genera and some mycotoxin producers. Early on we identified a negative correlation between the *R*. *gauvreaurii group* (a commensal genus involved in starch degradation and acetate, formate, and succinate production) and *Aspergillus* (the genus responsible of bovine aspergillosis) [7,60,61]. Later, we observed a positive correlation between *Aureobasidium* and *Lachnospiraceae* UCG-001 (Fig 5A), both of which are involved to ruminal digestion and VFAs production, respectively. In fact, according to Freitas *et al*. (2020), *Aureobasidium* is directly related to weight gain in bovines because of its ability to produce substances that improve digestion. For its part, *Lachnospiraceae* UCG-001, a normal constituent of the bovine intestinal microbiota, is involved in anti-inflammatory processes and the production of VFAs from polysaccharides [25,49]. However, in this study, we found that this genus had a reduced relative abundance in the *F*. *hepatica*-positive cattle, which suggests that it could also be happening with *Aureobasidium*. This raises the possibility that the loss of weight observed in cattle with fascioliasis is affected by the reduced abundance of commensal microorganisms associated with weight gain such as *Aureobasidium*. However, future studies should evaluate how *Fasciola* impacts on the functional products of commensal microorganisms and if the abundance of certain microorganisms is associated with weight loss. On the other hand, future studies should evaluate potential coinfections of *Fasciola* and other helminths that can impact these findings and unfortunately is a limitation in our study.

As far as we know, this study provides the first estimates of the impact of a liver helminth on the composition and relative abundance of intestinal microbial communities in bovines. On one side, our analysis reveals those cattle testing positive for *F*. *hepatica* had reduced diversity in both prokaryotic and eukaryotic microorganisms. However, the microbial composition is strongly affected by many factors, such as diet, age, geographical location, previous diseases, and even temporal variations in the same individual. Therefore, the changes found here are not necessarily a consequence of the presence of *Fasciola* only which highlights a limitation for our study. On the other hand, significant changes were found in the relative abundance of *F*. *hepatica* disease biomarkers, as observed in the reduction of some commensal taxa that have been previously reported as responsible for fermentation and digestion processes. The above could suggest a reduction in the ability to perform this function in cattle with fascioliasis. However, other approaches are needed to provide empirical evidence of associations between the reduction in the ability to digest and ferment plant material, and the weight loss observed in cattle with fascioliasis.

External factors such as other helminths may have implications on the abundance of microbial taxa [5,9]. However, once we evaluated coinfections, no significant differences were found between the abundance of commensal microorganisms in bovines with coinfections and those without coinfection at the time of sampling. The above indicates that, at least in this study, the alterations we observed, at a relative abundance level, were not probably caused by the presence of other nematodes and/or flatworms. Despite that, we suggest that future studies consider the use of naïve cattle to record each cattle’s infection pattern over time to ensure previous infections do not affect the results obtained. At the same time, all this would make it possible to provide an equivalent parasite load in all individuals and determine how it affects the composition and abundance of microorganisms in the infected bovines. Another limitation in our study is that we were not able to diagnose all the individuals using parasitological methods because the number of fecal eggs was often low. Eggs are only excreted by adult parasites (not those in the larval stages) and, considering the irregular oviposition of *F*. *hepatica*, this factor will need to be considered in future approaches. Lastly, future studies should consider a larger sample size to corroborate our findings. Overall, despite of the limitations herein highlighted, this study represents the first descriptive study towards the understanding of prokaryotic and eukaryotic communities inhabiting the gut ecosystems in bovines with and without *Fasciola* infection. Our findings provide an advance in the knowledge required to develop alternative treatments to antihelmintics to modulate microbial communities at the intestinal level. The effects of non-intestinal parasitic infections on species of veterinary interest are a little explored field; therefore, it is possible that our findings will be restricted to the *F*. *hepatica* helminth–host system. This highlights the need for further studies to corroborate our observations using other helminth parasites.

## Supporting information

S1 Fig Rarefaction curve for samples corresponding to the 16S analysis. (TIF)

S2 Fig Rarefaction curve for samples corresponding to the 18S analysis. (TIF)

S3 Fig Relative abundances of eukaryotes by 18S. (TIF)

S4 Fig Heatmaps of the most abundant families of the representative phyla of prokaryotes (B-D) and eukaryotes (A)
(TIF)

S5 Fig Differential abundance volcano plot of prokaryotic microorganisms between infection states. For ANCOM procedure, the clr (centered log ratio) table of ASVs grouped by genera was used, which was transformed to adjust to values from 0 to 1. The W value represents the number of times in which the hypothesis that the abundance of the microbial groups was the same in both infection states was rejected. A positive X axis means that a genus is more abundant in the *Fasciola*-negative group and a negative value of the X axis means that a genus is more abundant in the *Fasciola*-positive group. Genera with reject null-hypothesis> 95% were considered significant and are abbreviated as following: *Solobacterium* (SOL), *Paeniclostridium* (PAE), *Parabacteroides* (PAR) and *Allorhizobium-Neorhizobium-Pararhizobium-Rhizobium* (ANPR).
(PDF)

S6 Fig Differential abundance volcano plot of eukaryotic microorganisms between infection states. For ANCOM procedure, the clr (centered log ratio) table of ASVs grouped by genera was used, which was transformed to adjust to values from 0 to 1. The W value represents the number of times in which the hypothesis that the abundance of the microbial groups was the same in both infection states was rejected. A positive X axis means that a genus is more abundant in the *Fasciola*-negative group and a negative value of the X axis means that a genus is more abundant in the *Fasciola*-positive group. Genera with reject null-hypothesis> 95% were considered significant and are abbreviated as following: *Pseudozyma* (PSE), *Neocallimastix* (NEO), *Pilobolus* (PIL), *Apiospora* (API), *Cyllamyces* (CYL), *Leptosphaeria* (LEP), *Buxtonella* (BUX), *Cystobasidiomycetes_X* (CYS), *Rhodosporidium* (RHO), *Hyphozyma* (HYP), *Talaromyces* (TAL), *Thelebolus* (THE) and *Filobasidium* (FIL).
(PDF)

S7 Fig ALDEx2 effect size plot for Eukaryotic microorganisms between infection states. In the plot, red represents differentially abundant genera with p-value < 0.05; grey are genera that are abundant but not nondifferentially abundant, and black represents rare genera that are not differentially abundant. In the figure, the differentially abundant genera are abbreviated as *Pseudozyma* (PSE), *Talaromyces* (TAL), *Pichia* (PIC), *Filobasidium* (FIL), and *Cyperus* (CYP).
(TIF)

S1 Table Sociodemographic variables, coinfections and identification method of Fasciola in the analyzed samples. (XLSX)

S2 Table Filtered sequences. (CSV)

S3 Table ASVs table corresponding to 16S sequences by samples grouping the sequences taxonomically assigned to the same genus. (CSV)

S4 Table ASVs table corresponding to 18S sequences by samples grouping the sequences taxonomically assigned to the same genus. (CSV)

S5 Table Taxonomic assignment for the genus found by ANCOM. (XLSX)
