# Supplementary figures and images for: Describing the intestinal microbiota of Holstein Fasciola-positive and -negative cattle from a hyperendemic area of fascioliasis in central Colombia
Source: PLoS Negl Trop Dis. 2021 Aug 9;15(8):e0009658. doi: 10.1371/journal.pntd.0009658 (PMC8375995; doi:10.1371/journal.pntd.0009658)

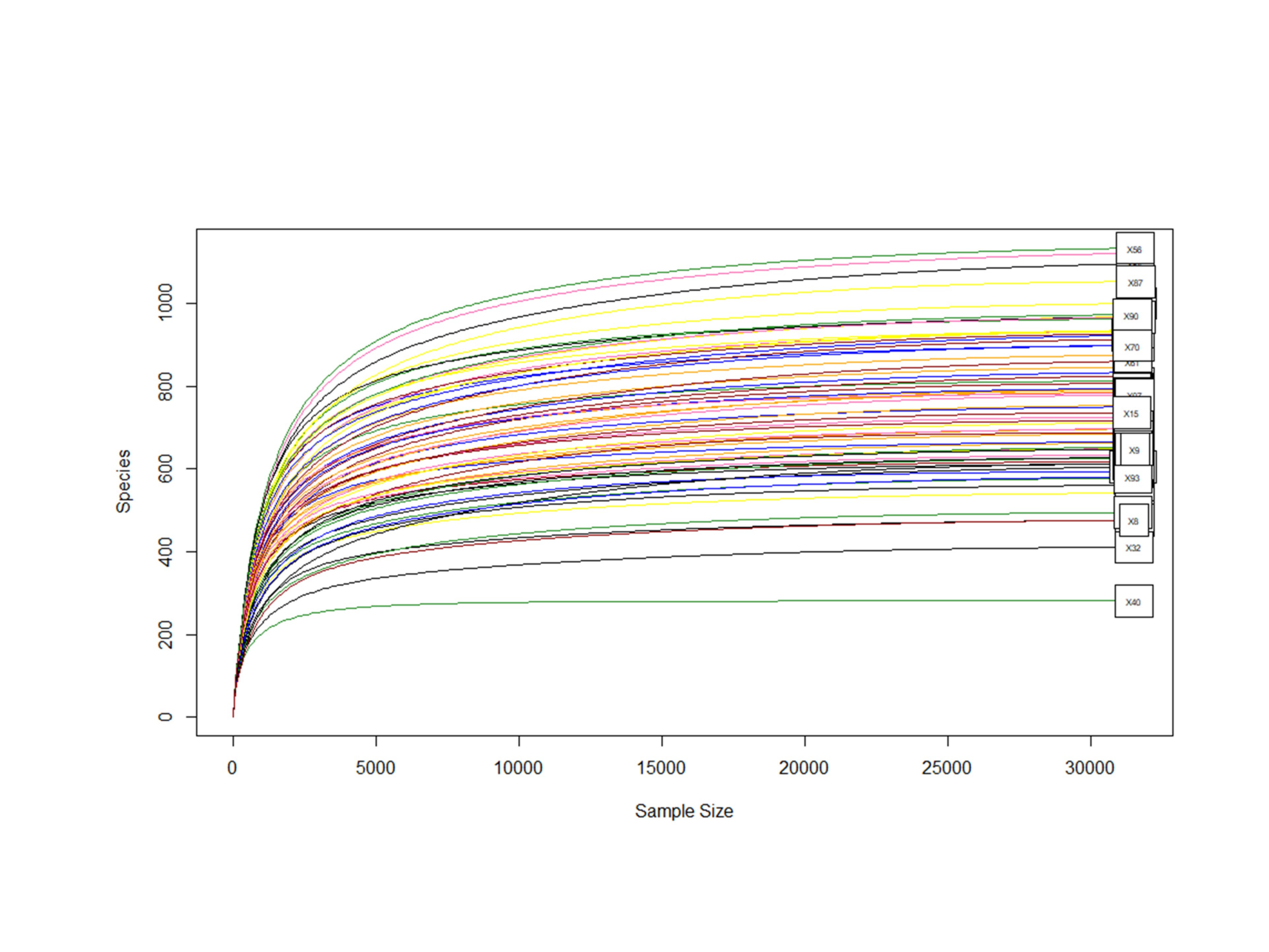

Supplement: S1 Fig — (TIF) [file pntd.0009658.s001.tif]

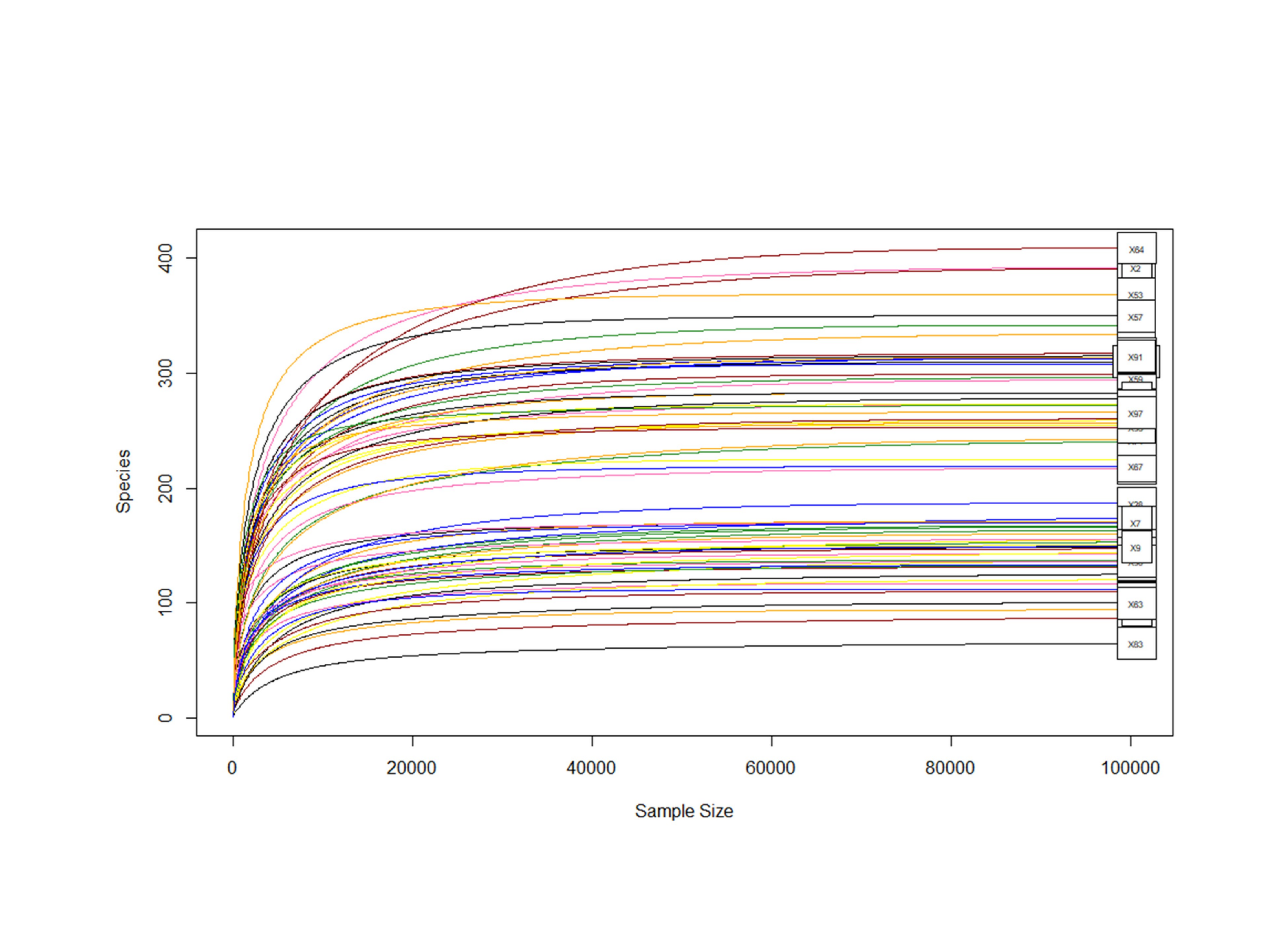

Supplement: S2 Fig — (TIF) [file pntd.0009658.s002.tif]

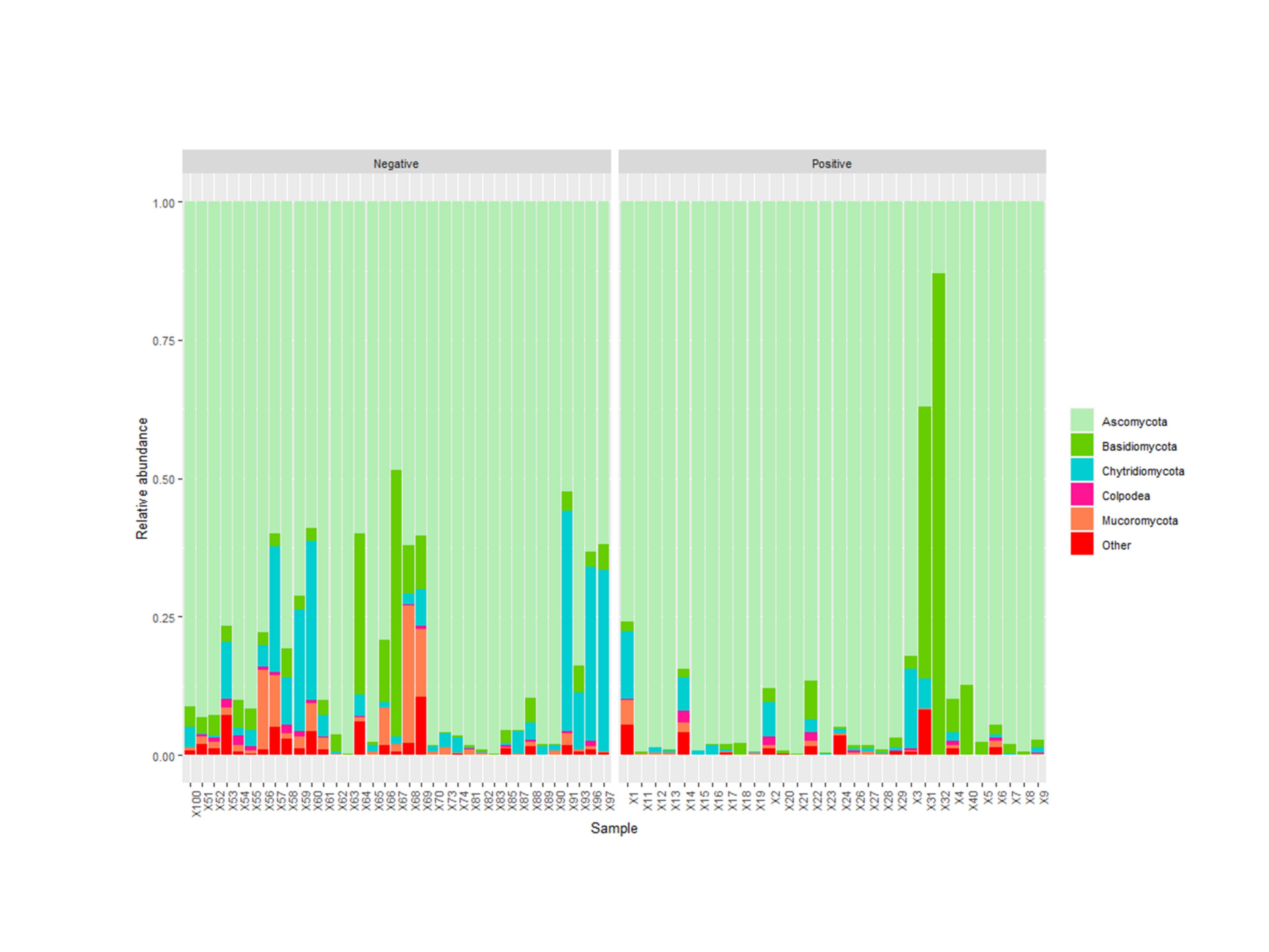

Supplement: S3 Fig — (TIF) [file pntd.0009658.s003.tif]

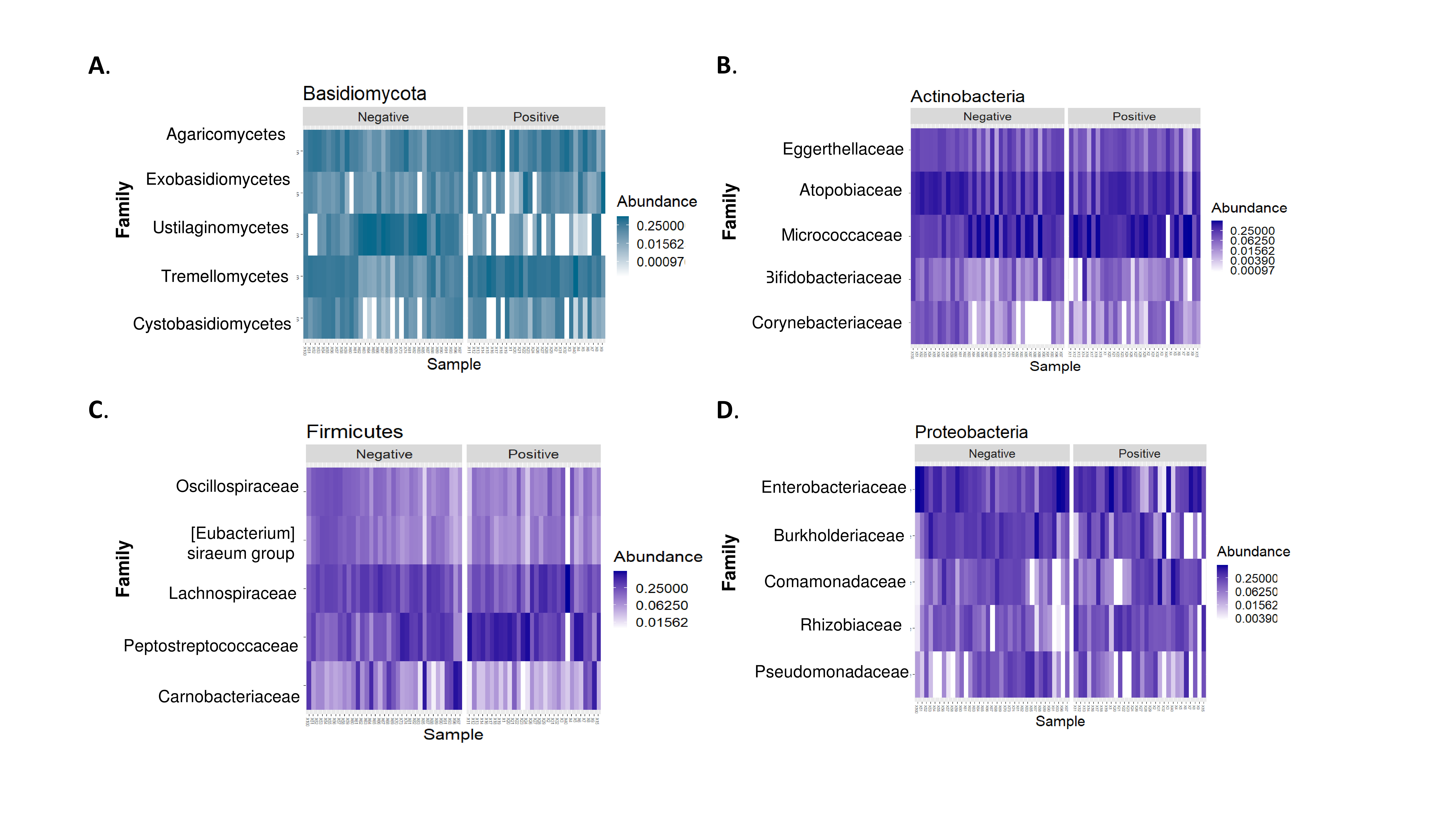

Supplement: S4 Fig — Heatmaps of the most abundant families of the representative phyla of prokaryotes (B-D) and eukaryotes (A) (TIF) [file pntd.0009658.s004.tif]

Structural zero    ● Yes    ● No

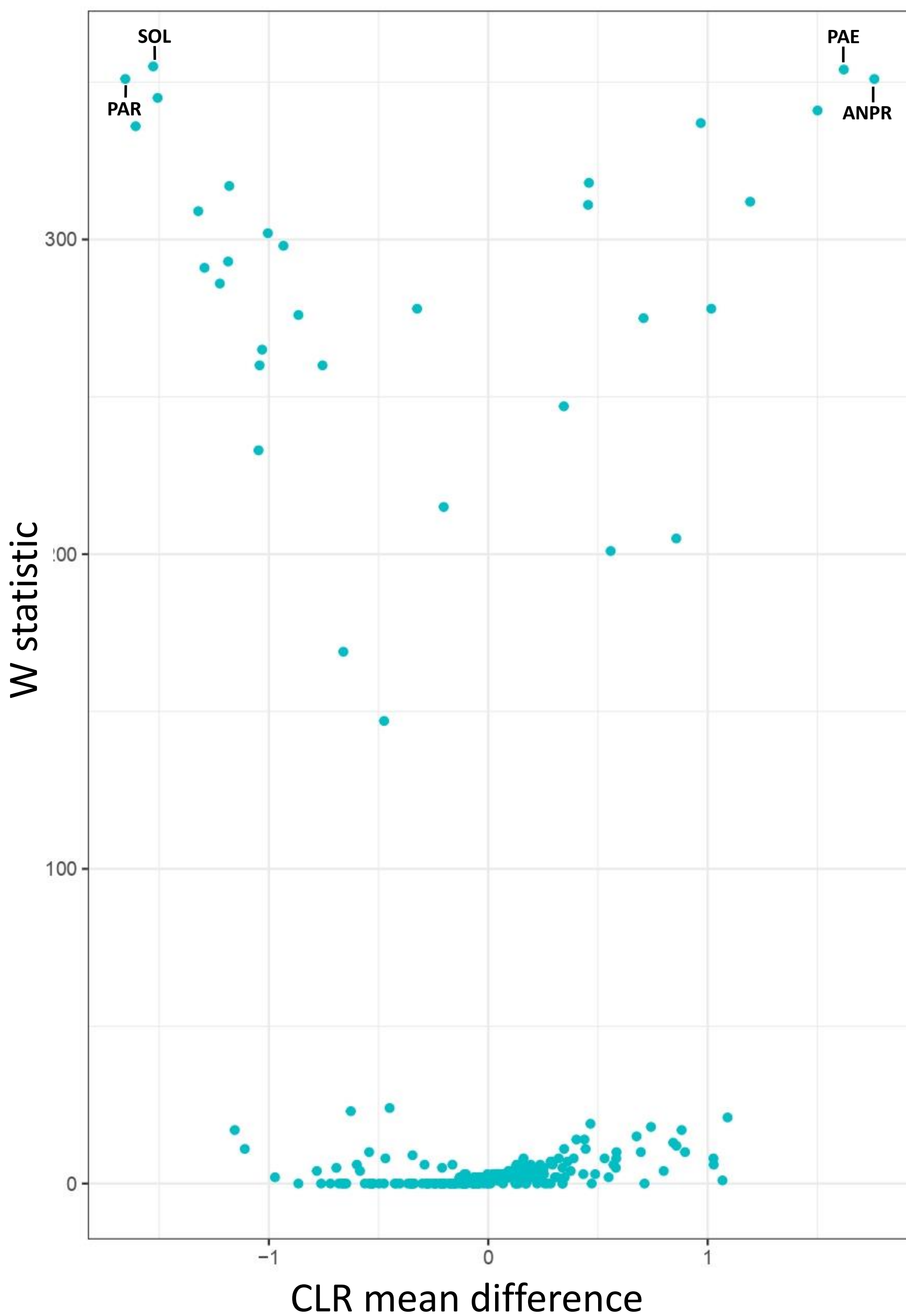

Supplement: S5 Fig — For ANCOM procedure, the clr (centered log ratio) table of ASVs grouped by genera was used, which was transformed to adjust to values from 0 to 1. The W value represents the number of times in which the hypothesis that the abundance of the microbial groups was the same in both infection states was rejected. A positive X axis means that a genus is more abundant in the Fasciola-negative group and a negative value of the X axis means that a genus is more abundant in the Fasciola-positive group. Genera with reject null-hypothesis> 95% were considered significant and are abbreviated as following: Solobacterium (SOL), Paeniclostridium (PAE), Parabacteroides (PAR) and Allorhizobium-Neorhizobium-Pararhizobium-Rhizobium (ANPR). (PDF) [file pntd.0009658.s005.pdf]

Structural zero    ● Yes    ● No

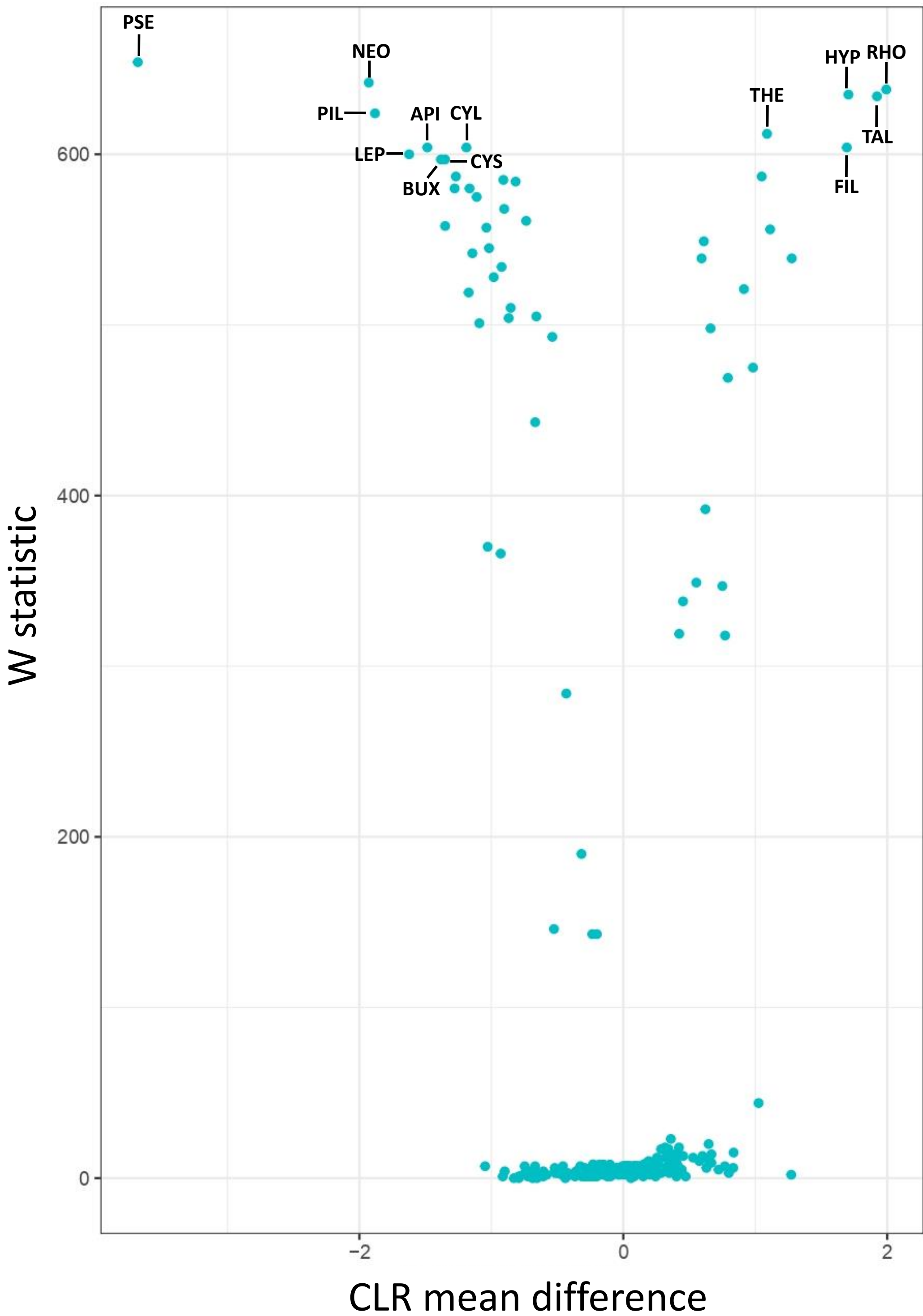

Supplement: S6 Fig — For ANCOM procedure, the clr (centered log ratio) table of ASVs grouped by genera was used, which was transformed to adjust to values from 0 to 1. The W value represents the number of times in which the hypothesis that the abundance of the microbial groups was the same in both infection states was rejected. A positive X axis means that a genus is more abundant in the Fasciola-negative group and a negative value of the X axis means that a genus is more abundant in the Fasciola-positive group. Genera with reject null-hypothesis> 95% were considered significant and are abbreviated as following: Pseudozyma (PSE), Neocallimastix (NEO), Pilobolus (PIL), Apiospora (API), Cyllamyces (CYL), Leptosphaeria (LEP), Buxtonella (BUX), Cystobasidiomycetes_X (CYS), Rhodosporidium (RHO), Hyphozyma (HYP), Talaromyces (TAL), Thelebolus (THE) and Filobasidium (FIL). (PDF) [file pntd.0009658.s006.pdf]

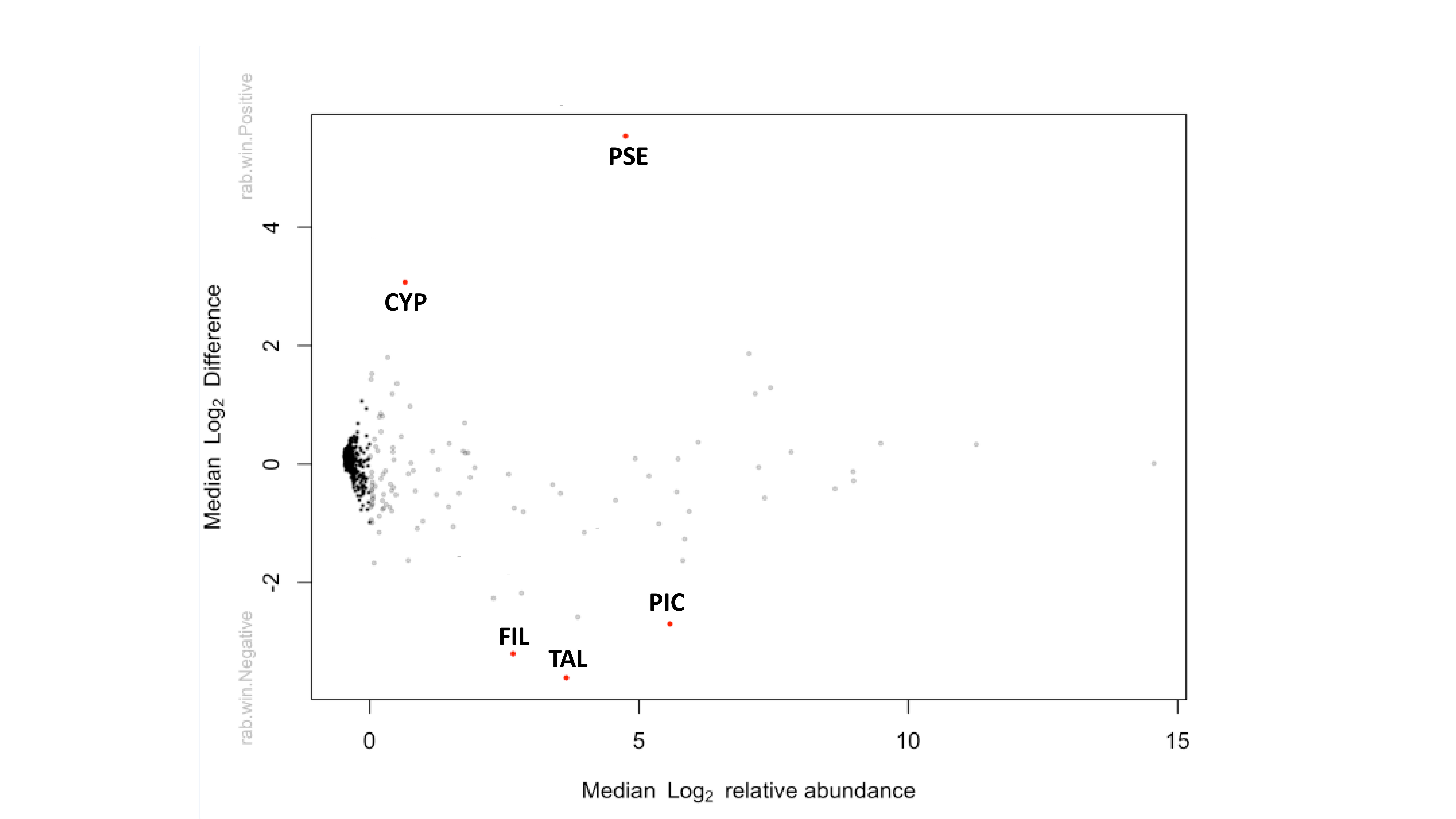

Supplement: S7 Fig — In the plot, red represents differentially abundant genera with p-value < 0.05; grey are genera that are abundant but not nondifferentially abundant, and black represents rare genera that are not differentially abundant. In the figure, the differentially abundant genera are abbreviated as Pseudozyma (PSE), Talaromyces (TAL), Pichia (PIC), Filobasidium (FIL), and Cyperus (CYP). (TIF) [file pntd.0009658.s007.tif]
